# Deriving Accurate Nocturnal Heart Rate, rMSSD and Frequency HRV from the Oura Ring

**DOI:** 10.3390/s24237475

**Published:** 2024-11-23

**Authors:** Tian Liang, Gizem Yilmaz, Chun-Siong Soon

**Affiliations:** Centre for Sleep and Cognition, Yong Loo Lin School of Medicine, National University of Singapore (NUS), Singapore 117549, Singapore; liang.tian@nus.edu.sg (T.L.); cs.soon@nus.edu.sg (C.-S.S.)

**Keywords:** heart rate variability, sleep, wearables, device validation

## Abstract

Cardiovascular diseases are a major cause of mortality worldwide. Long-term monitoring of nighttime heart rate (HR) and heart rate variability (HRV) may be useful in identifying latent cardiovascular risk. The Oura Ring has shown excellent correlation only with ECG-derived HR, but not HRV. We thus assessed if stringent data quality filters can improve the accuracy of time-domain and frequency-domain HRV measures. 92 younger (<45 years) and 22 older (≥45 years) participants from two in-lab sleep studies with concurrent overnight Oura and ECG data acquisition were analyzed. For each 5 min segment during time-in-bed, the validity proportion (percentage of interbeat intervals rated as valid) was calculated. We evaluated the accuracy of Oura-derived HR and HRV measures against ECG at different validity proportion thresholds: 80%, 50%, and 30%; and aggregated over different durations: 5 min, 30 min, and Night-level. Strong correlation and agreements were obtained for both age groups across all HR and HRV metrics and window sizes. More stringent validity proportion thresholds and averaging over longer time windows (i.e., 30 min and night) improved accuracy. Higher discrepancies were found for HRV measures, with more than half of older participants exceeding 10% Median Absolute Percentage Error. Accurate HRV measures can be obtained from Oura’s PPG-derived signals with a stringent validity proportion threshold of around 80% for each 5 min segment and aggregating over time windows of at least 30 min.

## 1. Introduction

Cardiovascular diseases (CVD) have become the global leading cause of death, accounting for over 10 million deaths worldwide annually [1]. Continuous monitoring of physiological signals such as heart rate (HR) contributes to better detection of abnormalities during free-living conditions. Higher HR, especially during sleep, is strongly associated with increased mortality and CVD risk [2]. On the other hand, lower heart rate variability (HRV), which is an indicator of altered autonomic nervous system activity [3], predicts higher CVD risk in population studies [4,5]. Long-term and large-scale monitoring of HR and HRV during sleep may contribute to more effective CVD prevention with earlier risk detection. However, the standard electrocardiogram (ECG) measurement used in clinical and research settings is neither scalable nor suitable for long-term use under free living conditions [6,7].

Commercially available sleep trackers with embedded photoplethysmography (PPG) sensors can support the long-term continuous acquisition of HR and HRV data with high temporal resolution. PPG is an established, non-invasive optical technique that can be used to estimate changes in blood volume coupled to heart rate [8]. The green, red, and infrared light sensors are commonplace in existing wrist- or finger-worn devices, as they are cost-efficient to implement and produce reliable results [6,9]. Wearable-derived HR and HRV show good agreement with reference measurements when taken during rest and sleep [10,11,12]. The Oura Ring, a finger-worn sleep tracker, is a validated device for measuring sleep and HR [13,14,15], and a potential candidate for long-term cardiovascular monitoring. The ring form factor is smaller and less obtrusive than wrist-wearables, and thus is well-suited for physiological measurements during sleep. Like most other wearables [16], Oura only provides time-domain HRV metrics (such as root mean square of successive differences, rMSSD). However, insufficient sleep disrupts cardiac autonomic balance by increasing sympathetic activity while decreasing parasympathetic activity [17,18,19], which is indexed by High Frequency HRV [20]. In the long run, this may result in CVD [5,21]. Hence, in addition to rMSSD, acquiring frequency-based HRV measures from wearables could provide a more comprehensive profile of cardiovascular well-being. However, despite showing high HR accuracy, a recent study [13] which evaluated a comprehensive suite of frequency-based HRV metrics from Oura data found only moderate agreement with concurrent reference ECG measures, falling short of acceptable accuracy.

A simple processing detail may have contributed to the poor correspondence in HRV measures between Oura and ECG. For each inter-beat-intervals (IBI) reading, Oura provides a validity rating based on their quality assessment filters (for details, see [10]). Following [10,13] accepted a 5 min segment of Oura IBI readings if at least 30% of readings within that time window were rated as valid. While this validity proportion threshold could still yield a relatively accurate measure of mean HR within a 5 min period, the impact on HRV accuracy is substantially more detrimental and deserves careful consideration. Variability metrics like rMSSD rely on the continuity of data, which are compromised by gaps left by missing or rejected IBI readings [22,23]. Spectral components can also be distorted by missing IBI, especially if up to 70% may be missing at a 30% validity proportion threshold, thus reducing the accuracy of frequency measures [24,25].

Hence, we propose a simple solution to improve the accuracy of HRV metrics from the Oura Ring: apply a more stringent validity proportion threshold. Stringent filtering reduces data gaps, ensuring that only epochs with sufficient valid IBIs to represent actual heart rate fluctuations are included, thus yielding more accurate and reliable HRV measures. Importantly, this reduces the likelihood of inaccurate or unstable HRV readings that may lead to undue concern for users. Our approach is also expected to improve HRV measures derived from other PPG-based wearables with ring or watch form factors, as they face similar challenges of increased susceptibility to transient artefacts related to poor contact or motion. During such periods, the total number of IBIs recorded may not be accurate due to invalid or missed IBIs, so a count-based approach to segment validity may not be appropriate [13]. Thus, we also used a different definition of validity proportion based on duration, i.e., the sum of valid IBIs divided by the time window.

Here, we showed that a more stringent validity proportion threshold of 80% can indeed provide accurate HRV measures of rMSSD and HF (compared against ECG). However, a higher validity proportion threshold also results in a higher data rejection rate, which needs to be taken into consideration [26]. Thus, we compared the accuracy of HR and HRV measures and data rejection rates at 80%, 50%, and 30% validity thresholds to evaluate the practicality of increasing validity proportion thresholds.

## 2. Materials and Methods

### 2.1. Participants and Protocol

Data from two previously published studies with concurrent ECG (SOMNOmedics, GmbH, Randersacker, Germany) and Oura Ring (Gen 3, Oura Ring Inc., Oulu, Finland) recordings [27,28] were combined for the current analyses. Participants were not clinically diagnosed with pre-existing sleep, neurological, or psychiatric disorders; not experiencing excessive daytime sleepiness (Epworth Sleepiness Scale < 11, [29]); not taking wake-promoting medication; habitually sleep more than 5 h per night; and have a body mass index (BMI) <35 kg/m^2^. Anthropometric measurements like height, weight, waist circumference, and office blood pressure (BP) were taken during a daytime briefing prior to their sleep session. Participants slept according to their habitual bed- and wake-times in a sleep laboratory while polysomnography (PSG), ECG, and Oura Ring data were collected simultaneously during their sleep. Informed consent was obtained during the briefing for both studies. The Institutional Review Board of the National University of Singapore approved the protocols, which were compliant with the Declaration of Helsinki.

For both studies, a study night’s recordings were included if they passed quality control checks. For Study 2, only recordings from the first night were included, as the second night involved a sleep disruption protocol, which is expected to affect HR and HRV measurements [30]. After excluding 28 participants from Study 1 and 23 from Study 2, 114 participants were included in the analyses (68 from Study 1; 50 males; 28.0 ± 15.8 years old). Participants were further divided into two age groups: younger (20–44 years old; N = 92; 45.7% male) versus older (45–68 years old; N = 22; 36.4% male) to examine whether HR and HRV measurement accuracies differed between younger and older participants [31].

### 2.2. Devices

**Figure 1:**
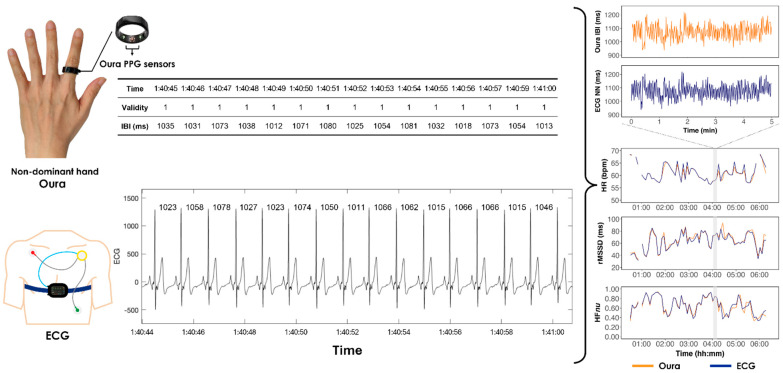
Diagram showing placement of devices and data samples before filtering. The right panel shows 5 min NN/IBI timeseries and derived HR and HRV values for one night.

ECG data were collected using the ECG electrodes of SOMNOtouch devices (SOMNOmedics, GmbH, Randersacker, Germany). The Oura Ring (Oura Ring Inc., Oulu, Finland) estimates IBI using PPG signals (sampling rate 250 Hz) collected via multiple infrared (900 nm) light sensors. A real-time moving average filter is applied to the raw PPG signals to locate local maxima and minima in order to compute IBI, after which IBI normalcy is assessed through median filters [32]. In both studies, participants wore Oura Rings (Generation 3) on their non-dominant hand, as shown in Figure 1.

### 2.3. Data Analysis

**Figure 2:**
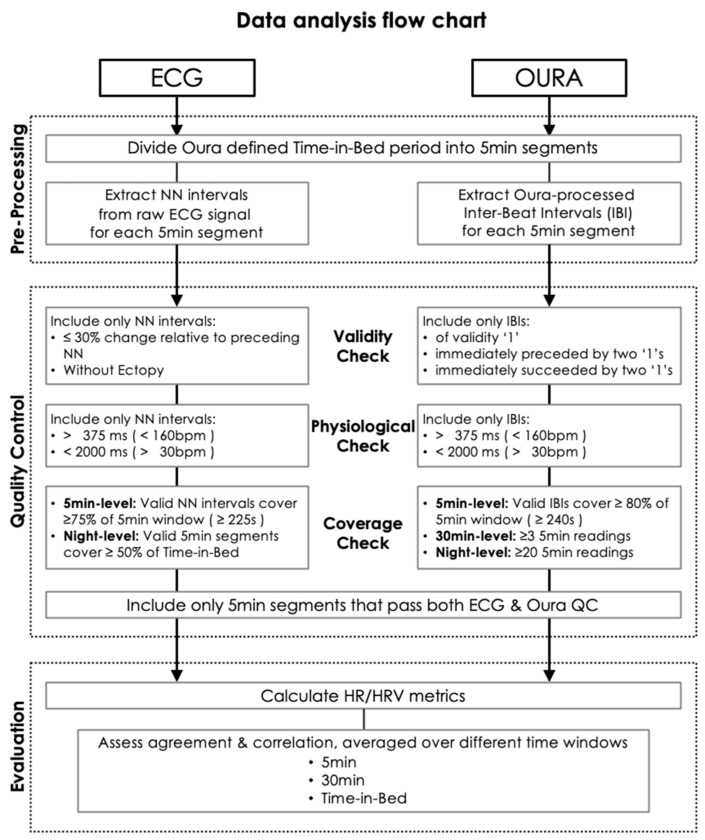
Flowchart showing ECG NN and OURA IBI processing pipelines. A stringent validity proportion threshold of 80% was applied to Oura IBI data.

ECG waveform analyses were conducted in MATLAB (R2021b; The Math Works, Inc., Natick, MA, USA). Oura IBI processing, HR and HRV calculation, and statistical comparisons were performed in RStudio (version 2023.12.0+369). As the Oura Ring’s efficacy as a standalone device for cardiovascular monitoring during sleep is being evaluated, for the current analyses, the start and end of sleep periods were defined using Oura-assessed bed- and wake times, which have been shown to be highly accurate when compared against PSG [33,34]. The Time-In-Bed period (TIB) was divided into 5 min segments for comparing HR and HRV metrics derived from ECG normal-to-normal (NN) and Oura IBI signals. Before further analyses, both time series were checked for: (1) validity of each NN or IBI reading; (2) physiological plausibility during sleep; and (3) validity proportion for each 5 min segment (Figure 2).

#### 2.3.1. Oura IBI

Raw PPG signals first underwent Oura’s internal processing to derive IBI readings. A validity rating was provided for each IBI reading, based on a 5-point ordinal scale from 0 to 4. Ratings other than ‘1’ indicated unreliable readings, so only IBIs rated ‘1’ were retained. Additionally, following [10], valid IBIs were retained only if two immediately preceding and succeeding IBIs were also ‘1’. Next, physiologically implausible IBIs during sleep were removed (IBI < 375 ms or HR > 160 bpm; IBI > 2000 ms or HR < 30 bpm; [13,35]). Finally, we evaluated the impact of different validity proportion thresholds on the degree of correlation and agreement between Oura Ring- and ECG-derived HR and HRV measures at 80%, 50%, and 30%. To ensure representative coverage of the night, we only accepted nights where at least twenty 5 min epochs survived the validity proportion threshold. Reference [13] accepted a 5 min segment if at least 30% of the number of readings within the 5 min were rated as valid. However, invalid readings can be unusually long or short, sometimes due to artefacts like poor contact or motion, so the IBI count may not be a reliable indicator of the actual number of heartbeats. This issue also precluded beat-to-beat matching of Oura IBI and ECG NN. Thus, we adopted a duration-based definition of validity proportion: the fraction of time accounted for by valid IBIs within a time window, i.e., for a 5 min segment to pass a validity proportion threshold of 80%, the sum of valid IBIs must be at least 240 s.

#### 2.3.2. ECG NN

R-peaks were automatically identified using the QRS detector from the PhysioNet Cardiovascular Signal Toolbox [36], with the following validity and plausibility criteria: (1) lower and upper limits of peak-to-peak duration were set to 375 ms and 2000 ms; (2) a maximum acceptable change in peak-to-peak duration between consecutive peaks of 30%. Misidentified R-peaks were deleted manually in a custom-made Graphic User Interface. No R-peak additions were performed. The NN interval time series was checked for abnormal patterns and any clustering in Poincaré plots suggestive of ectopy. Coverage checks were performed at the night and 5 min segment levels: (1) only 5 min segments in which at least 150 NN-intervals passed the above criteria (equivalent to 50% at 60 bpm) were accepted; and (2) only nights with at least 50% of 5 min segments accepted were retained.

### 2.4. HR and HRV Metrics

A 5 min window is conventionally used for short-term HRV assessment [37,38], and allows for comparisons across devices and studies. (Although we use the term HRV for consistency, the derived metric actually indicates pulse rate variability, as PPG does not directly measure the heart’s electric signatures.) Only 5 min segments that passed both ECG and Oura IBI quality control checks were included for further analyses. Mean HR and rMSSD were calculated for Oura IBI and ECG-NN for each 5 min segment. HF HRV was calculated using the Lomb-Scargle periodogram [39,40] via the Lomb package in R [41]. Per convention [38], HF power was calculated by taking the area under the NN periodogram for frequency ranges of 0.15–0.40 Hz. To facilitate comparison, HF values were normalized to the sum of HF and LF for the respective 5 min segments, resulting in HF*nu* values that ranged from 0 to 1 normalized units [42].

Finally, to assess correspondence over larger time windows, we averaged multiple 5 min Oura and ECG segments to obtain 30 min and nightly aggregates. To ensure representativeness, only 30 min windows containing at least three 5 min segments surviving the validity proportion threshold, and night-level windows with at least twenty such 5 min segments, were included for analyses.

### 2.5. Statistical Analyses

In addition to Pearson’s correlation coefficient (r), the agreement between ECG- and Oura-derived metrics were also assessed using the Concordance Correlation Coefficient (CCC), which evaluates how far the linear relationship between two variables deviates from the line of perfect concordance (i.e., y = x; [43]). Similarly to Pearson’s r, CCC values range from 0 to 1, with higher values indicating better correlation. The systemic errors between ECG and Oura IBI-based measures were evaluated using Bland–Altman plots and limits of agreement analysis [44].

Lastly, Mean Absolute Error (MAE), Mean Absolute Percentage Error (MAPE), and Median Absolute Percentage Error (MdAPE) for all HR/HRV metrics were used to evaluate discrepancies between Oura and ECG at the individual participant level. This was carried out for readings at the 5 min level, from which the 30 min and night level values were derived. As there is no clear consensus on which error metric to report for HR/HRV analyses, we report all 3 with median (interquartile range, IQR) values due to slightly skewed distributions (Supplementary Material, Table S2). MAPE and MdAPE values < 10% were considered acceptable for both HR [45], and HRV [15] measures. The formulae for the error metrics are as follows:

$$\mathrm{Mean}\;\mathrm{Absolute}\;\mathrm{Error}\;(\mathrm{MAE})=\frac{1}{n}\sum_{i=1}^{n}\left|H_{Oura}-H_{ECG}\right|$$

$$\mathrm{Mean}\;\mathrm{Absolute}\;\mathrm{Percentage}\;\mathrm{Error}\;(\mathrm{MAPE})=\frac{1}{n}\sum_{i=1}^{n}\frac{\left|H_{Oura}-H_{ECG}\right|}{H_{ECG}}$$

$$\mathrm{Median}\;\mathrm{Absolute}\;\mathrm{Percentage}\;\mathrm{Error}\;(\mathrm{MdAPE})=median(\sum_{i=1}^{n}\frac{\left|H_{Oura}-H_{ECG}\right|}{H_{ECG}})$$

where H stands for HR, rMSSD, or HF*nu.*

### 2.6. Data Retention Rates at Different Validity Proportion Thresholds

Data included in this study were minimally affected by artefacts, as the collection period was during sleep and in a controlled laboratory environment. However, under free-living conditions, where data collection can be affected by various factors, the 80% validity proportion threshold may result in significant data loss, compromising data representativeness and feasibility [26]. Thus, out of practical necessity, other than the accuracy of HR and HRV metrics, it is important to also consider the effect of validity proportion thresholds on data retention rates. Here, we also report the proportion of 5 min segments that are retained at 80%, 50%, and 30% validity proportion thresholds, with the expectation that these may be lower under free living conditions.

## 3. Results

Table 1 summarizes participant demographics and sleep characteristics. The median BMI and blood pressure for both younger and older groups fell within the healthy range. Compared to younger participants, older participants had earlier bed- and wake times and shorter TIB.

### 3.1. Performance Evaluation

**Figure 3:**
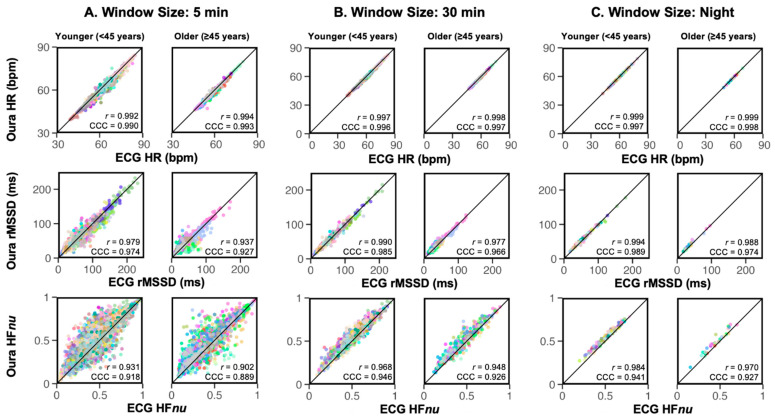
Scatter plots showing correlations between Oura IBI-derived measures and ECG-derived measures separated by age group for different window sizes: (**A**) 5 min, (**B**) 30 min and (**C**) night levels. Validity proportion threshold was set at 80%. Every colour represents one participant within the age group.

We first present results for a validity proportion threshold of 80%. Overall, HR and HRV showed high correlations of 0.9 and above for both Pearson’s r and CCC (Figure 3A–C), with the only exception being CCC for 5 min HF*nu* for the older group, at 0.889 (Figure 3A). Nevertheless, it was clear that at the 5 min level, individual HRV readings could differ substantially between Oura and ECG. However, these discrepancies were reduced with larger window sizes, with all points clustering very closely to the line of equality for all night-level measures. HRV measures, especially HF*nu*, appear to be more susceptible to random noise at the 5 min level. To demonstrate that higher validity proportion thresholds lead to better correlation, we repeated the analyses at 95% threshold (Figure S3) only for the 5 min window. Across all metrics and age groups, very high correlations of at least 0.9 were obtained. Most notably, the correlations between Oura and ECG HR were virtually 1.00. The HF*nu* correlations were also greater than 0.93, indicating very high levels of similarity between the Oura- and ECG-derived values.

**Figure 4:**
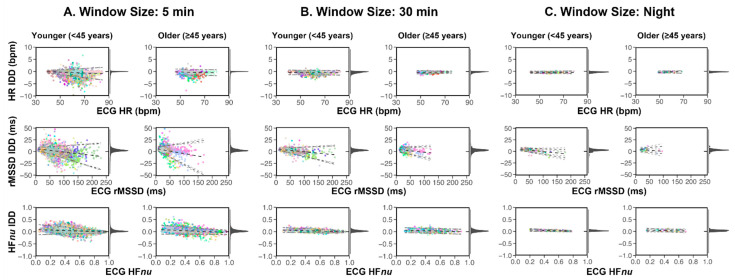
Bland–Altman plots showing the agreement between Oura IBI-derived measures and ECG-derived measures (reference) separated by age for different window sizes: (**A**) 5 min, (**B**) 30 min, and (**C**) Night. IDD (inter-device difference) was defined as ECG measures subtracted from the respective Oura measures. The IDD distributions, plotted on the right of each Bland–Altman plot, showed a tight cluster around 0. Results are shown for 80% validity proportion threshold. Every colour represents one participant within the age group.

We then investigated the presence and extent of systemic errors between Oura and ECG through Bland–Altman plots (Figure 4). Overall, mean biases were small across all measures (HR 0.42–0.64 bpm; rMSSD 2.50–3.79 ms; HF*nu* 0.03). There was a slight proportional bias for rMSSD, as seen in the downward slope across all window sizes. HR and HF did not show proportional biases. Across all metrics, wider limits of agreement were observed at the 5 min level relative to the 30 min and Night levels. Older participants had lower HR and rMSSD ranges than younger participants, but no major differences in IDD were observed between the two groups for any metric.

**Figure 5:**
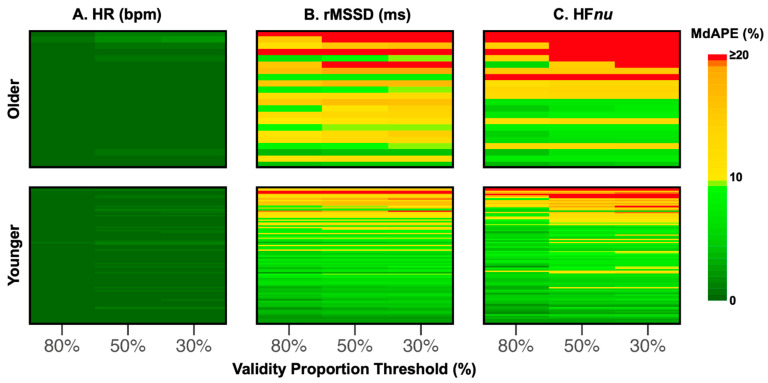
Heatmaps showing participant-level MdAPE values at different validity proportion thresholds for HR/HRV measures. Each row represents one individual across all validity proportion thresholds. Within each age group, participants were sorted by the mean MdAPE across the validity proportion thresholds. While errors were very low across the board for HR (dark green), many participants had unacceptable rMSSD and HF*nu* MdAPE (>10%, yellow and red), especially those in the older group.

MdAPE was calculated for each participant’s 5 min level HR/HRV readings at different validity proportion thresholds and visualized as heatmaps (Figure 5; MAE and MAPE showing similar trends are shown in Supplementary Materials Figures S1 and S2), with every individual row as one participant. Within each heatmap per age group, the individual rows were arranged in descending order of mean MdAPE across all validity proportion thresholds.

As expected, HR was the best performing metric, with very low errors for every participant, even at a relaxed threshold of 30% (Figure 5A). In contrast, for both rMSSD and HF*nu*, some participants had MdAPEs of 20% or higher (red). Even though there was a clear effect of validity proportion threshold, with the lowest error rates at 80%, HRV MdAPE remained above the acceptable threshold of 10% (yellow and red) for many participants at this stringent threshold. While most younger participants had acceptably low HRV MdAPE of <10%, more than half of the older participants had HRV MdAPE >10% (yellow and red).

### 3.2. Lower Data Retention with Higher Validity Proportion Threshold

Even though our analyses showed that a high validity proportion threshold could improve HR and HRV metrics for the Oura Ring, this came at the cost of rejecting many 5 min segments of IBI readings, as seen in Table 2. At an 80% validity proportion threshold, about 30–35% of the data were rejected. Data retention was even worse at the 95% level, where despite very high correlation (Supplementary Material, Figure S3), only about 30–45% of the data remained. Such poor coverage could undermine the representativeness of the derived metrics.

## 4. Discussion

This study assessed the accuracy of Oura IBI-derived HR/HRV metrics against ECG-derived ones at different validity proportion thresholds. We showed that, using a stringent validity proportion threshold of 80%, HR, rMSSD, and HF*nu* derived from the Oura Ring in 5 min windows showed very high correlations with respective reference ECG metrics, even for older participants. That being said, higher discrepancies were found at the individual level between Oura and ECG for both rMSSD and HF*nu*, especially at more lenient validity proportion thresholds, and for older participants in particular. In contrast, less stringent validity proportion thresholds had almost no impact on HR. The concordance between Oura and ECG HR/HRV measures further improved when averaged over 30 min epochs, and substantially more so across the whole night of sleep. No evidence of substantial bias was detected at larger time windows, implying that highly accurate HRV measures can be obtained from finger PPG signals, as long as random measurement noise at the 5 min level was cancelled out. Table 3 summarizes the main results of the current study in comparison with recent studies comparing wearable HR/HRV against ECG, which reported correlations and mean biases [10,13,46,47,48]. Overall, correlations between wearable and ECG HR were very high across all studies, with very low absolute mean bias. For studies that reported rMSSD, correlations were high, and mean bias was generally low except for [13]. Compared against the only other study that reported frequency-based HRV metrics [13], we had much higher correlation and lower bias. 

### 4.1. High Correlation in HR/HRV Between Oura and ECG

As expected, we found high correlation and agreement between Oura- and ECG-derived HR [13,15,48,49]. Here, we further showed that more stringent quality filtering of the Oura data produced more accurate HR with reduced errors. Importantly, more substantial improvements were seen for Oura HRV measures with higher validity proportion thresholds (Figure 5 and Supplementary Material, Table S1). At the 80% validity proportion threshold, both Pearson’s and Concordance correlations were tending towards 1 for HR and mostly above 0.9 for HRV metrics, much better than previously reported [10,13]. These results were achieved for the shortest acceptable time window for assessing HRV, 5 min, and even better agreement was seen for larger time windows, with neither *r* value nor CCC below 0.9 even for the frequency-based HF*nu*.

### 4.2. Less Accurate 5 Min HRV Measures for Older Participants

Even though Oura-derived HR/HRV metrics showed high concordance with ECG, to qualify as a heart health monitoring device, it is also important to assess the error rates for individual participants. The current analyses considered whether accurate HR/HRV metrics could also be obtained for older participants aged 45 years and above, as age has been shown to affect the accuracy of HRV measures from PPG signals [31,50,51,52]. We observed relatively high MdAPE (>10%) for 5 min HRV readings of older participants (11.36 (6.88) for rMSSD and 11.82 (8.25) for HF*nu*, as median (IQR), reported in Table S2). Since there was no difference in systematic errors between younger and older participants (as shown with Bland–Altman plots in Figure 4), the observed difference in error metrics is more likely due to individual variability rather than a consistent bias between devices.

In Figure 5, individual participants were sorted by average MdAPE across all measures, i.e., the same row represented the same participant across the heatmaps of all three measures. We observed that participants with high MdAPE in one HRV measure also tended to have higher errors in the other. The exact reasons for this observation in our sample remained unclear as we had access to processed Oura IBI but not the full raw PPG waveforms. One possibility is that the specific wave shapes of the PPG signals from which IBI are estimated are less temporally precise for some individuals. This could be due to reduced skin perfusion or arterial stiffness, which is likely more prevalent in older participants [53,54]. Age-related changes in the PPG waveform, such as smoothing and rounding of systolic peaks [50,55], may interfere with accurate systolic peak detection and IBI estimation. While our findings did not suggest there were systematic errors for the Oura Ring, it remains unclear whether the high error rates for some elderly participants were also associated with undetected cardiovascular conditions or other issues like poor ring fit due to age-related loss of soft tissue.

Even at an extremely high validity proportion threshold of 95%, there were substantial discrepancies between Oura and ECG HRV metrics (Supplementary Material, Figure S3) indicative of inherent limitations in the accuracy of single 5 min HRV measures. In contrast, such lack of temporal precision would not increase HR error by much, as it has little impact on the number of heartbeats detected. Thus, the HR MdAPE for all participants remained below 5%, even at more lenient validity proportion thresholds. HR is an overall average of heart beats within a minute, whereas HRV reflects the variation in intervals between successive heart beats and is more sensitive to the accuracy and continuity of the interval values. Compared to the temporal precision of the ECG R-peaks, there is likely to be some noise in the IBI estimation from PPG waveforms. The discrepancy may be greater for some older participants if the PPG waveform is distorted due to ageing, resulting in higher error values. However, as we only had access to the Oura-defined IBI values and not the raw PPG sensor data, we are unable to further evaluate the accuracy and validity ratings of the Oura IBI values.

Fortunately, despite the relatively high error rate at the 5 min level, the discrepancies between Oura and ECG HRV measures were substantially reduced when aggregated over longer time windows, without showing substantive biases.

### 4.3. Aggregate Oura HRV Measures over Longer Durations to Improve Accuracy

The 5 min window is conventionally used for short-term HRV assessment [37,38] and may reflect momentary autonomic fluctuations [56]. For long-term heart health monitoring, the accuracy of longer HRV measurements, especially during the night, is also important, since it can be predictive of poor cardiovascular outcomes [57,58]. Here, we found a higher concordance and correlation at larger window sizes (compared to results from 5 min), which is in line with the existing literature [59,60]. In Figure 3, concordance improved from the 5 min to 30 min time windows and lay very close to the line of perfect equality between Oura and ECG at the night level, regardless of age group. Only HF*nu* showed a slight over-estimation of 0.03–0.04 nu in Oura data for both younger and older participants. This implies that errors seen at the 5 min level may largely be due to random measurement noise rather than systematic errors in the Oura Ring PPG-derived IBI. The higher error rate at the 5 min level should be taken into consideration when trying to assess HRV fluctuations through the night using PPG-based consumer wearables. The likelihood of measurement noise may increase in free-living conditions and in individuals with cardiovascular disease, hindering the reliability of HRV assessment. More precise investigation of individual variability in PPG waveforms and its impact on derived IBI values is needed to reduce such measurement errors.

### 4.4. Data Rejection Costs of Further Increasing Validity Proportion Threshold

Alternatively, the validity proportion threshold could be increased further to improve HRV accuracy at the 5 min level. At a 95% validity proportion threshold (Supplementary Material, Figure S3), the 5 min HRV accuracy was comparable to averaging over 30 min windows at an 80% threshold (*r* = 0.935–0.988, CCC = 0.926–0.983; Figure 3). However, more than half of the 5 min segments (younger: 67.2%; older: 55.3%) for younger and older participants could not meet the 95% threshold (Table 2). Under free-living conditions, the rejection rates could be even higher, potentially compromising the representativeness of the remaining HRV values. Further, nights with less than 50% of 5 min windows passing quality checks were excluded from our analyses (Figure 2), i.e., at a 95% validity proportion threshold, most nights would be excluded. Thus, we do not recommend increasing the validity proportion threshold beyond 80%.

### 4.5. Limitations

Our findings suggest that reliable HRV measures from a PPG-based wearable device are achievable with an 80% validity proportion threshold, and averaged over at least 30 min of data. However, these recommendations need to be tested under free living conditions for different demographics. Firstly, it should be noted that our data were acquired within an ideal sleep laboratory environment, and more artefacts may be expected under free-living conditions. As mentioned above, this could result in lower data retention rates, potentially leaving less than 50% usable data over the whole sleep period at an 80% validity proportion threshold. Secondly, while we considered the effect of age and found high correlations even for our older participants as a group, their individual error rates tended to be higher than those of younger participants. Considering that our sample included only healthy participants, higher error rates would be expected in older participants with various health conditions. For example, we did not directly examine the effects of anomalies like ectopic beats. We excluded ectopic beats from ECG data, but, without directly analyzing PPG waveforms, could only rely on Oura’s IBI validity rating to exclude them. Future studies should evaluate the feasibility and accuracy of different validity proportion thresholds and window length in diverse demographic samples under free living conditions.

## 5. Conclusions

In this study, we evaluated whether accurate HRV measures during sleep can be derived from PPG-based signals acquired using a wearable in a ring form factor. We showed that, at more stringent validity proportion thresholds, the HR/HRV metrics derived from Oura IBI showed high correlation and agreement with the ECG reference measure regardless of age group or window size. Nevertheless, the trade-off between accuracy and data retention must be carefully considered. In addition, 5 min Oura HRV error rates were relatively high for older participants, but higher accuracy with minimal biases can be achieved when averaged over longer time windows. Overall, our results suggest that the Oura Ring has the potential to be a long-term heart health monitoring device that can provide accurate HR and HRV measures, provided HRV measures are derived using a stringent validity proportion threshold of around 80%, and 5 min readings are averaged over a longer time window of at least 30 min.

## Figures and Tables

**Table 1:** Participant demographics and sleep characteristics, in median (IQR).

	Units	Younger (<45 Years)		Older (≥45 Years Old)		Mann-Whitney U Test *p*-Value
**Demographics**						
Number of participants		92	[42 males]	22	[8 males]	
Age	years	25.0	(10.3)	56.5	(12.0)	*p* < 0.001
BMI	kg/m²	22.1	(3.2)	24.3	(3.0)	*p* = 0.019
Office SBP	mmHg	110.0	(16.0)	117.0	(19.5)	*p* = 0.059
Office DBP	mmHg	70.0	(9.0)	75.0	(17.0)	*p* = 0.169
**Sleep Characteristics (Oura)**						
Time-in-Bed	hours	7.52	(1.10)	7.38	(0.73)	*p* = 0.980
Bed-time	hh:mm	00:45	(1.57 h)	23:55	(1.46 h)	*p* = 0.005
Wake-time	hh:mm	08:18	(1.96 h)	07:02	(1.56 h)	*p* = 0.009
Sleep Efficiency	%	90.0	(7.0)	87.0	(9.0)	*p* = 0.089

**Table 2:** Data retention rates at different validity proportion thresholds.

Validity Proportion Threshold	Retention Rate (%)	
	Younger	Older
*Initial 5 min segment count*	*14,073*	*3485*
30%	93.3	95.2
50%	87.0	89.4
80%	66.7	72.3
95%	32.8	44.7

**Table 3:** Summary of findings from wearable HR/HRV validation studies.

Publication	Devices	Setting	Window Size	Study Sample (Age ± SD)	* Correlations (*r*)	* Mean Bias
Kinnunen et al., 2020 [10]	Oura Ring Gen 2 (PPG, finger) Somnologica/Faros 90/Faros 180 (ECG, Reference)	Free living, S	5 min *	N = 49 (31.6 ± 11.8)	HR = 0.996 rMSSD = 0.980	HR = −0.63 bpm rMSSD = −1.20 ms
Benedetti et al., 2021 [46]	FitBit ChargeHR (PPG, wrist) Morpheus Home Portable PSG (ECG, Reference)	Free living, S	1 min *	N = 25 (22.4 ± 3.0)	HR < 100 bpm HR = 0.84 HR > 100 bpm HR = 0.35	HR = −0.66 bpm
Nuuttila et al., 2021 [47]	Polar Vantage V2 (PPG, wrist) Polar H10 (ECG, Reference)	Free living, S	5 min *	N = 29 (36.0 ± 7.0)	HR = 0.998 ln(rMSSD) = 0.963	HR = 0.70 bpm ln(rMSSD) = 0.17 ms
Cao et al., 2022 [13]	Oura Ring Gen 3 (PPG, finger) Shimmer 3 (ECG, Reference)	Free living, S	5 min * Night	N = 46 (32.3 ± 6.4)	HR = 0.993 rMSSD = 0.915 SDNN = 0.518 AVNN = 0.825 LF (absolute) = 0.424 HF (absolute) = 0.627 LF/HF ratio = 0.354	HR = −0.44 bpm rMSSD = −14.97 ms SDNN = −0.96 ms AVNN = −13.39 ms LF (absolute) = 23.61 ms² HF (absolute) = 30.23 ms² LF/HF ratio = −0.11
Henriksen et al., 2022 [48]	Oura Ring Gen 2 (PPG, finger) Actiheart 4 (ECG, Reference)	Free living, S	Night *	N = 21 (33.0 ± 14.0)	RHR = 0.900	RHR = −1.00 bpm
Current paper	Oura Ring Gen 3 (PPG, finger) SOMNOtouch (ECG, Reference)	In-lab, S	5 min * 30 min Night	Younger N = 92 (27.4 ± 6.5) Older N = 22 (58.0 ± 6.9)	Younger HR = 0.992 rMSSD = 0.979 HF*nu* = 0.931 Older HR = 0.994 rMSSD = 0.937 HF*nu* = 0.902	Younger HR = −0.64 bpm rMSSD = 2.50 ms HF*nu* = 0.03 Older HR = −0.42 bpm rMSSD = 3.79 ms HF*nu* = 0.03

* For each study, only results for this time window is shown. S—Sleep; W—Wake; SDNN—Standard Deviation of NN intervals; AVNN—Average of NN intervals; RHR—Resting Heart Rate; ln(rMSSD)—Natural log of rMSSD.

## Data Availability

Data and source code are available upon request.

## Supplementary Materials

The following supporting information can be downloaded at: https://www.mdpi.com/article/10.3390/s24237475/s1, Figure S1. MAPE HR/HRV heat maps for (A) HR, (B) rMSSD, and (C) HF*nu*; Figure S2. MAE HR/HRV heat maps for (A) HR, (B) rMSSD, and (C) HF*nu*; Figure S3. Scatter plots showing the relationship between Oura and ECG-derived HR/HRV metrics at 95% validity proportion threshold. Pearson’s r and Concordance Correlation Coefficient (CCC) are specified at the bottom right-hand corner;; Table S1. Descriptive statistics for Oura IBI and ECG-derived HR/HRV metrics and mean bias; Table S2. Numerical values for correlations and error metrics (MAE, MAPE, MdAPE). Top to bottom in ascending window size: 5 min, 30 min, and Night level.
