# Supplementary material for: Deriving Accurate Nocturnal Heart Rate, rMSSD and Frequency HRV from the Oura Ring
Source: Sensors (Basel). 2024 Nov 23;24(23):7475. doi: 10.3390/s24237475 (PMC11644394; doi:10.3390/s24237475)
Supplement: Supplementary file 1 [file sensors-24-07475-s001.zip › sensors-3248262-supplementary.pdf]

sensors

# Supplementary Materials

# Deriving Accurate Nocturnal Heartrate, rMSSD and Frequency HRV from the Oura Ring

**Tian Liang, Gizem Yilmaz * and Chun-Siong Soon**

Centre for Sleep and Cognition, Yong Loo Lin School of Medicine, National University of Singapore (NUS), Singapore 117549, Singapore; liang.tian@nus.edu.sg (T.L.); cs.soon@nus.edu.sg (C.-S.S.)
* Correspondence: gizem.yilmaz@nus.edu.sg

**Table S1.** Descriptive statistics for Oura IBI and ECG-derived HR/HRV metrics and mean bias

| Metrics | Window Size | ECG: Mean (SD) | | Oura: Mean (SD) | | Bias: Mean (SD) | |
|---|---|---|---|---|---|---|---|
| | | Younger | Older | Younger | Older | Younger | Older |
| HR | 5min | 60.72 (7.15) | 57.65 (5.56) | 60.29 (7.07) | 57.36 (5.54) | -0.64 (1.35) | -0.42 (0.89) |
| | 30min | 60.62 (6.87) | 57.54 (5.42) | 60.19 (6.86) | 57.26 (5.45) | -0.43 (0.49) | -0.28 (0.30) |
| | Night | 60.21 (6.39) | 57.26 (4.79) | 59.79 (6.39) | 56.97 (4.85) | -0.42 (0.24) | -0.29 (0.18) |
| rMSSD | 5min | 54.33 (27.97) | 33.92 (20.33) | 56.66 (26.53) | 36.67 (19.11) | 2.50 (7.47) | 3.79 (8.59) |
| | 30min | 54.58 (27.05) | 34.36 (19.56) | 56.94 (25.72) | 37.14 (18.79) | 2.48 (5.11) | 3.88 (5.67) |
| | Night | 56.97 (26.19) | 35.05 (18.63) | 59.20 (24.74) | 38.08 (17.98) | 2.23 (3.11) | 3.03 (2.85) |
| HF*nu* | 5min | 0.52 (0.20) | 0.45 (0.21) | 0.56 (0.20) | 0.48 (0.20) | 0.03 (0.07) | 0.03 (0.09) |
| | 30min | 0.52 (0.17) | 0.45 (0.17) | 0.56 (0.16) | 0.49 (0.16) | 0.03 (0.04) | 0.04 (0.05) |
| | Night | 0.53 (0.12) | 0.45 (0.12) | 0.57 (0.11) | 0.49 (0.12) | 0.03 (0.02) | 0.04 (0.03) |

**Table S2.** Numerical values for correlations and error metrics (MAE, MAPE, MdAPE). Top to bottom in ascending window size: 5min, 30min, and Night level.

| Metric | Validity Proportion | Window Size | MAE: Median (IQR) | | MAPE: Median (IQR) | | MdAPE: Median (IQR) | | r | | CCC | |
|---|---|---|---|---|---|---|---|---|---|---|---|---|
| | | | Younger | Older | Younger | Older | Younger | Older | Younger | Older | Younger | Older |
| HR | 80% | 5min | 0.45 (0.21) | 0.30 (0.23) | 0.74 (0.41) | 0.51 (0.44) | 0.16 (0.12) | 0.14 (0.11) | 0.992 | 0.994 | 0.990 | 0.993 |
| | 50% | | 0.66 (0.46) | 0.41 (0.29) | 1.04 (0.73) | 0.68 (0.54) | 0.29 (0.30) | 0.20 (0.28) | 0.981 | 0.986 | 0.977 | 0.983 |
| | 30% | | 0.70 (0.51) | 0.42 (0.28) | 1.10 (0.76) | 0.72 (0.58) | 0.31 (0.35) | 0.22 (0.35) | 0.977 | 0.984 | 0.972 | 0.980 |
| rMSSD | 80% | 5min | 3.94 (2.25) | 4.54 (1.71) | 8.42 (6.51) | 15.36 (7.11) | 7.00 (6.23) | 11.36 (6.88) | 0.979 | 0.937 | 0.974 | 0.927 |
| | 50% | | 4.78 (2.37) | 5.75 (1.93) | 9.79 (7.34) | 18.40 (8.24) | 7.23 (6.34) | 12.34 (7.81) | 0.972 | 0.909 | 0.967 | 0.892 |
| | 30% | | 5.21 (2.94) | 6.28 (2.58) | 10.77 (7.13) | 20.44 (10.83) | 7.52 (6.41) | 13.15 (7.29) | 0.968 | 0.889 | 0.963 | 0.867 |
| HF*nu* | 80% | 5min | 0.05 (0.02) | 0.06 (0.02) | 11.89 (7.62) | 19.13 (15.68) | 5.74 (4.45) | 11.82 (8.25) | 0.931 | 0.902 | 0.918 | 0.889 |
| | 50% | | 0.06 (0.03) | 0.07 (0.03) | 14.86 (10.21) | 22.92 (19.77) | 7.37 (5.10) | 13.22 (12.68) | 0.903 | 0.870 | 0.884 | 0.847 |
| | 30% | | 0.06 (0.03) | 0.07 (0.03) | 15.24 (11.48) | 24.70 (19.29) | 7.76 (5.24) | 13.24 (13.70) | 0.893 | 0.855 | 0.872 | 0.828 |

| Metric | Validity Proportion | Window Size | MAE: Median (IQR) | | MAPE: Median (IQR) | | MdAPE: Median (IQR) | | r | | CCC | |
|---|---|---|---|---|---|---|---|---|---|---|---|---|
| | | | Younger | Older | Younger | Older | Younger | Older | Younger | Older | Younger | Older |
| HR | 80% | 30min | 0.39 (0.28) | 0.27 (0.19) | 0.63 (0.47) | 0.44 (0.37) | 0.49 (0.45) | 0.37 (0.43) | 0.997 | 0.998 | 0.996 | 0.997 |
| | 50% | | 0.57 (0.49) | 0.34 (0.32) | 0.91 (0.86) | 0.60 (0.60) | 0.74 (0.76) | 0.49 (0.59) | 0.994 | 0.995 | 0.989 | 0.991 |
| | 30% | | 0.59 (0.52) | 0.36 (0.30) | 0.96 (0.84) | 0.63 (0.65) | 0.75 (0.78) | 0.49 (0.60) | 0.993 | 0.995 | 0.988 | 0.990 |
| rMSSD | 80% | 30min | 3.34 (1.65) | 3.91 (1.62) | 6.95 (6.37) | 13.45 (7.11) | 6.54 (6.22) | 12.60 (6.65) | 0.990 | 0.977 | 0.985 | 0.966 |
| | 50% | | 4.02 (2.20) | 4.75 (2.04) | 7.75 (7.31) | 15.40 (8.48) | 6.61 (6.74) | 13.53 (7.54) | 0.987 | 0.954 | 0.981 | 0.933 |
| | 30% | | 4.10 (2.29) | 5.02 (3.44) | 8.34 (7.49) | 17.94 (12.29) | 6.84 (6.73) | 14.91 (7.00) | 0.986 | 0.941 | 0.980 | 0.914 |
| HF*nu* | 80% | 30min | 0.04 (0.02) | 0.05 (0.02) | 7.50 (5.76) | 12.87 (8.98) | 5.83 (6.18) | 8.92 (10.01) | 0.968 | 0.948 | 0.946 | 0.926 |
| | 50% | | 0.04 (0.03) | 0.05 (0.03) | 8.59 (6.35) | 14.04 (14.79) | 6.82 (6.55) | 10.14 (13.58) | 0.955 | 0.919 | 0.922 | 0.880 |
| | 30% | | 0.04 (0.03) | 0.06 (0.04) | 8.65 (7.53) | 14.77 (13.96) | 6.81 (7.56) | 10.30 (12.44) | 0.951 | 0.909 | 0.915 | 0.862 |

| Metric | Validity Proportion | Window Size | MAE: Median (IQR) | | MAPE: Median (IQR) | | MdAPE: Median (IQR) | | r | | CCC | |
|---|---|---|---|---|---|---|---|---|---|---|---|---|
| | | | Younger | Older | Younger | Older | Younger | Older | Younger | Older | Younger | Older |
| HR | 80% | Night | 0.38 (0.27) | 0.26 (0.24) | 0.60 (0.50) | 0.43 (0.44) | 0.60 (0.50) | 0.43 (0.44) | 0.999 | 0.999 | 0.997 | 0.998 |
| | 50% | | 0.58 (0.55) | 0.34 (0.36) | 0.97 (0.91) | 0.61 (0.71) | 0.97 (0.91) | 0.61 (0.71) | 0.998 | 0.998 | 0.992 | 0.993 |
| | 30% | | 0.62 (0.55) | 0.34 (0.34) | 1.02 (0.97) | 0.62 (0.73) | 1.02 (0.97) | 0.62 (0.73) | 0.997 | 0.997 | 0.990 | 0.992 |
| rMSSD | 80% | Night | 3.06 (2.05) | 3.41 (2.02) | 6.04 (6.89) | 12.50 (8.26) | 6.04 (6.89) | 12.50 (8.26) | 0.994 | 0.988 | 0.989 | 0.974 |
| | 50% | | 3.44 (2.49) | 4.68 (2.95) | 6.51 (7.34) | 14.88 (10.87) | 6.51 (7.34) | 14.88 (10.87) | 0.993 | 0.976 | 0.986 | 0.952 |
| | 30% | | 3.67 (2.62) | 4.86 (3.70) | 7.06 (7.96) | 17.61 (12.58) | 7.06 (7.96) | 17.61 (12.58) | 0.992 | 0.970 | 0.985 | 0.940 |
| HF*nu* | 80% | Night | 0.03 (0.02) | 0.04 (0.04) | 5.25 (5.70) | 8.65 (12.70) | 5.25 (5.70) | 8.65 (12.70) | 0.984 | 0.970 | 0.941 | 0.927 |
| | 50% | | 0.03 (0.03) | 0.05 (0.03) | 6.52 (7.57) | 10.21 (13.58) | 6.52 (7.57) | 10.21 (13.58) | 0.974 | 0.938 | 0.905 | 0.863 |
| | 30% | | 0.03 (0.04) | 0.06 (0.03) | 6.74 (7.93) | 11.54 (12.93) | 6.74 (7.93) | 11.54 (12.93) | 0.968 | 0.928 | 0.891 | 0.841 |

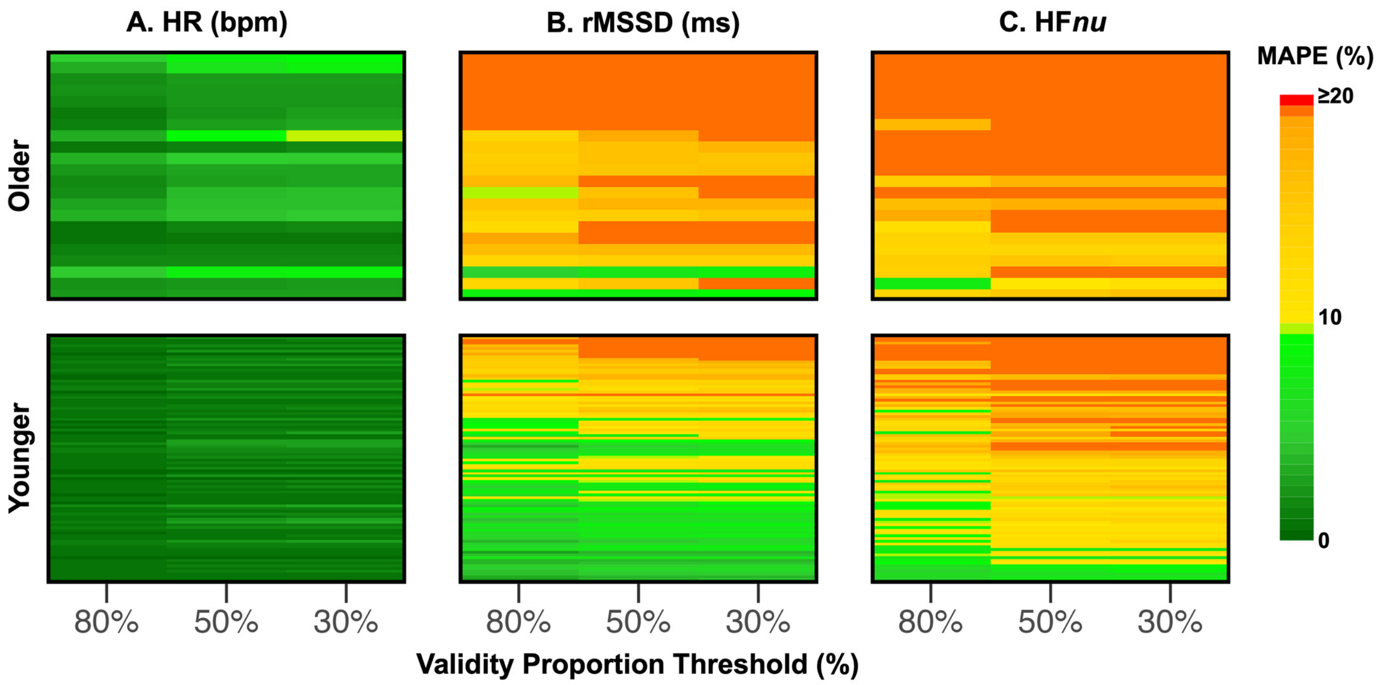


**Figure S1.** MAPE HR/HRV heat maps for (A) HR, (B) rMSSD, and (c) HFnu

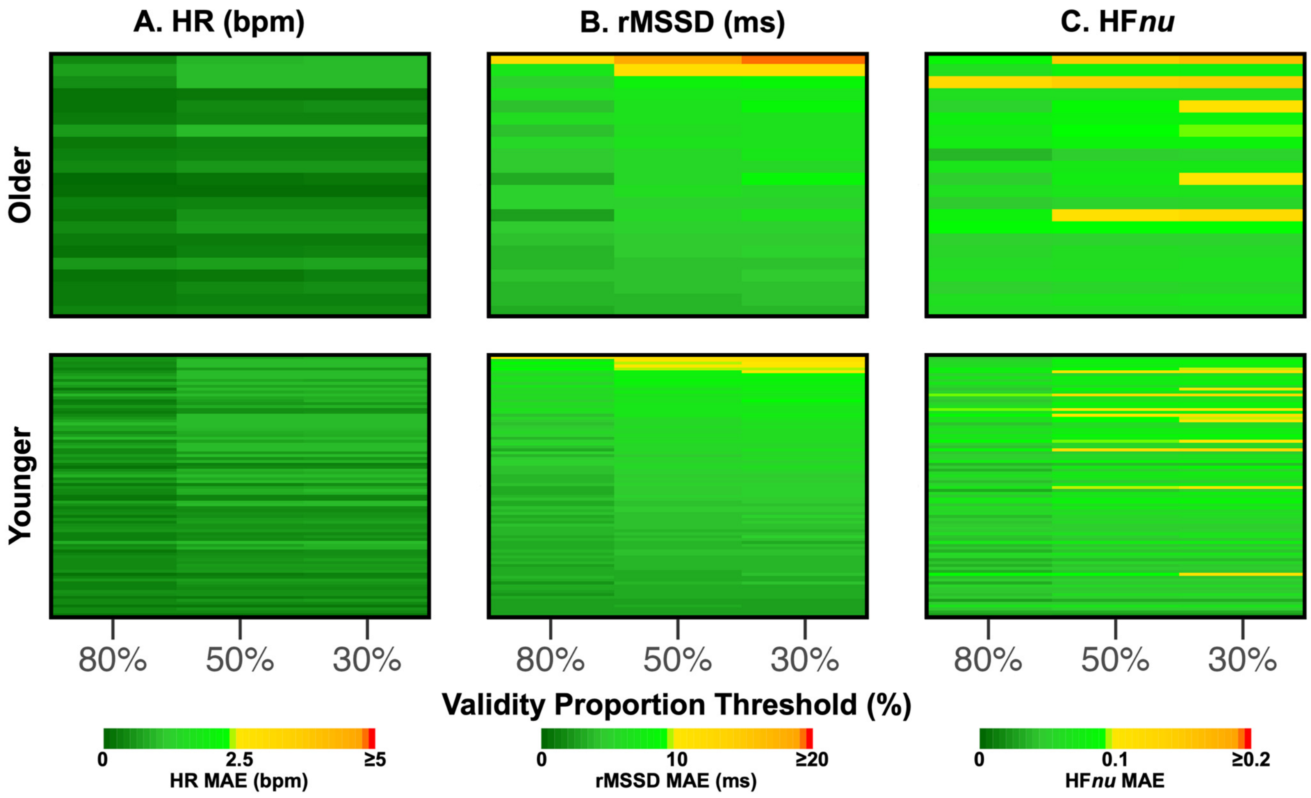


**Figure S2.** MAE HR/HRV heat maps for (A) HR, (B) rMSSD, and (c) HFnu

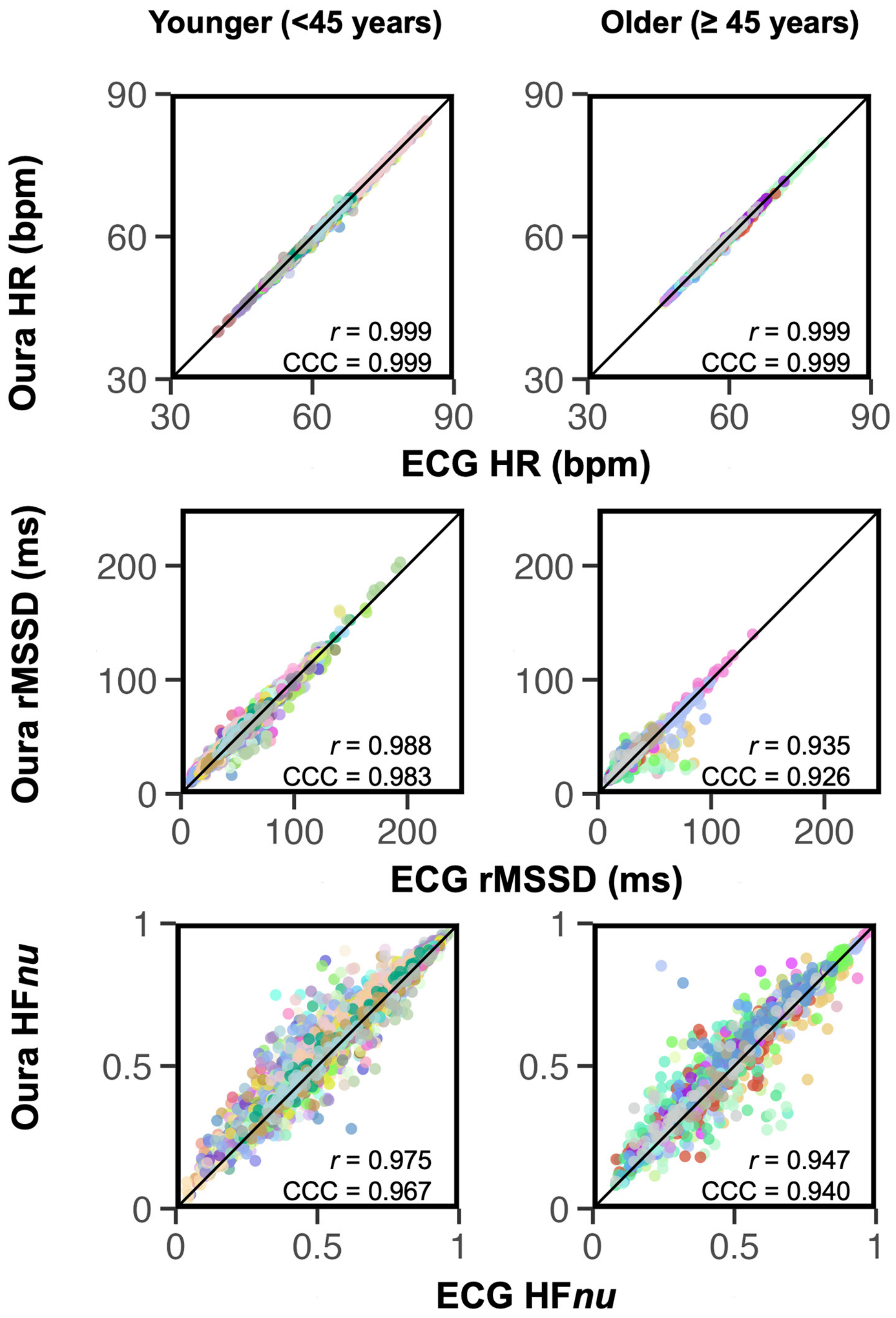


**Figure S3.** Scatter plots showing the relationship between Oura and ECG-derived HR/HRV metrics at 95% validity proportion threshold. Pearson's r and Concordance Correlation Coefficient (CCC) are specified at the bottom right-hand corner.
